# Elucidating information processing in primate basal ganglia circuitry: a novel technique for pathway-selective ablation mediated by immunotoxin

**DOI:** 10.3389/fncir.2013.00140

**Published:** 2013-09-03

**Authors:** Masahiko Takada, Ken-Ichi Inoue, Daisuke Koketsu, Shigeki Kato, Kazuto Kobayashi, Atsushi Nambu

**Affiliations:** ^1^Systems Neuroscience Section, Primate Research Institute, Kyoto UniversityInuyama, Japan; ^2^Division of System Neurophysiology, Department of Physiological Sciences, National Institute for Physiological Sciences, The Graduate University for Advanced StudiesOkazaki, Japan; ^3^Department of Molecular Genetics, Institute of Biomedical Sciences, Fukushima Medical University School of MedicineFukushima, Japan

**Keywords:** basal ganglia, hyperdirect pathway, information processing, immunotoxin, lentivirus, vectors, gene transfer, primates

## Abstract

Employing a neuron-specific retrograde gene-transfer vector (NeuRet vector), we have recently developed a novel technique that achieves pathway-selective ablation in the primate brain. This technique is mediated by immunotoxin (IT) and eliminates a neuronal population that constitutes a particular pathway, leaving other pathways intact. By means of this technique, we have made an attempt to remove the hyperdirect pathway selectively from basal ganglia circuitry. The hyperdirect pathway links the motor cortex to the subthalamic nucleus (STN) directly and plays a crucial role in motor control. After electrical stimulation in the motor cortex, triphasic responses consisting of an early excitation, an inhibition, and a late excitation are usually elicited in the internal pallidal segment (GPi). Several pieces of pharmacophysiological evidence imply that the early excitation may be derived from the hyperdirect pathway. In our experiments, the NeuRet vector expressing human interleukin-2 receptor α-subunit was injected into the STN of macaque monkeys. Then, IT injections were performed into the supplementary motor area (SMA). When single neuron activity in the GPi was recorded in response to the SMA stimulation, it was found that the early excitation was significantly reduced with neither the inhibition nor the late excitation affected. The spontaneous firing rate and pattern of GPi neurons remained to be altered. This clearly indicates that IT-mediated tract targeting successfully eliminated the hyperdirect pathway with spontaneous activity of STN neurons unaffected. The electrophysiological findings were histologically confirmed by retrograde and anterograde neuronal labeling. The overall data define that the motor cortically driven early excitation in GPi neurons is conveyed through the hyperdirect pathway. The IT-mediated pathway-selective ablation technique will provide a powerful tool for elucidating information processing in various neural networks.

## INTRODUCTION

To know about a variety of higher brain functions systematically, it is essential to elucidate the architecture of complex and elaborate neural networks. For clarifying the functional role of a given pathway, it is an effective way to explore behavioral and physiological changes due to ablation of a neuronal population that constitutes the target pathway. Neuronal targeting mediated by immunotoxin (IT) has been established in mice as a genetic method for eliminating a specific neuron group from a certain neural network (Kobayashi et al., 1995; Sano et al., 2003; Yasoshima et al., 2005). Recently, it has been revealed that the use of modified glycoprotein of rabies virus for preparing a pseudotyped lentiviral vector based on human immunodeficiency virus type 1 (HIV-1) can enhance the efficiency of gene transfer through retrograde transport of the vector (Kato et al., 2007, 2011a). This property of the pseudotyped lentiviral vector largely allows for gene transfer into cell bodies of neurons that are located remote from the injection site of the vector. For IT-mediated removal of a particular pathway, the highly efficient retrograde gene-transfer vector was produced to express human interleukin-2 receptor α-subunit (IL-2Rα), a receptor molecule for the recombinant IT, in neuronal cell bodies via retrograde transport of the vector. In mice receiving injection of the IL-2Rα-expressing vector into the striatum, IT injection into the thalamus successfully resulted in selective removal of the thalamostriatal pathway (Kato et al., 2011b).

**FIGURE 1:**
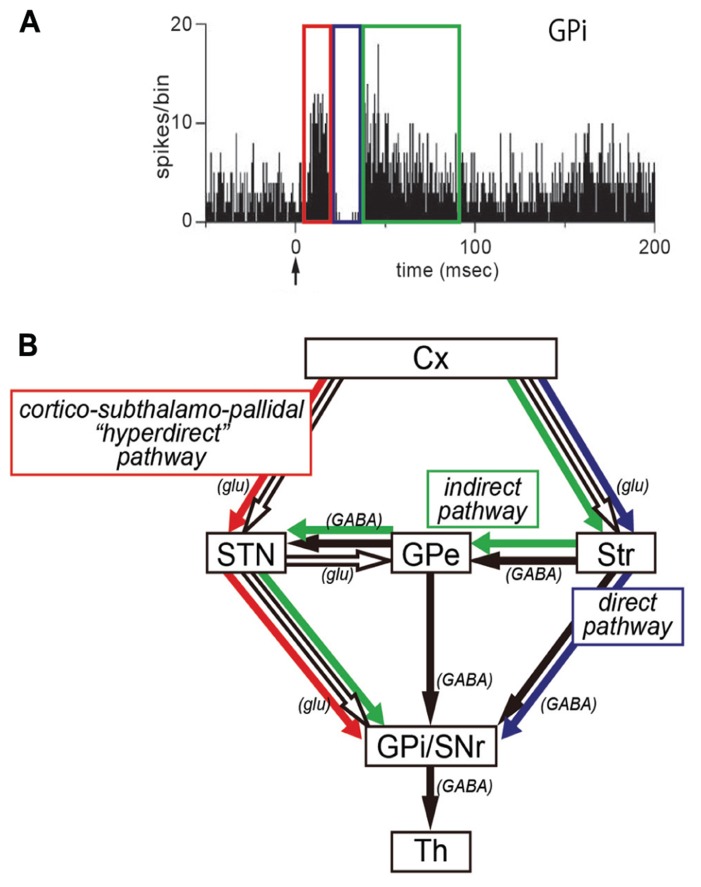
**(A)** Peri-stimulus time histogram (PSTH; bin width of 1 ms, summed for 100 stimulus trials) showing triphasic responses of a neuron in the internal segment of the globus pallidus (GPi). Electrical stimulation in the motor cortex was given at time = 0 (arrow). Note that an early excitation (red), an inhibition (blue), and a late excitation (green) are derived respectively from the hyperdirect, direct, or indirect pathway depicted in **(B)**. **(B)** Three pathways of the basal ganglia. Cx, cerebral cortex; glu, glutamate; GPe, external segment of the globus pallidus; GPi, internal segment of the globus pallidus; SNr, substantia nigra pars reticulata; STN, subthalamic nucleus; Str, striatum; Th, thalamus.

In our recent work, we have applied the IT-mediated pathway-selective elimination technique to the primate brain, because the use of non-human primates as animal models is critical for investigating higher brain functions. Employing the nigrostriatal dopamine pathway as a test system, we have first established the basic methodology with a neuron-specific retrograde gene-transfer vector (NeuRet vector) that has newly been developed with improved neuron specificity (Kato et al., 2011c). Next, an attempt has been made to eliminate the cortico–subthalamic “hyperdirect” pathway selectively from basal ganglia circuitry in macaque monkeys (Inoue et al., 2012). The subthalamic nucleus (STN) receives major input from the motor cortex and, in turn, sends output to the internal segment of the globus pallidus (GPi), a main output station of the basal ganglia (Hartmann-von Monakow et al., 1978; Mink and Thach, 1993; Parent and Hazrati, 1995; Mink, 1996; Nambu et al., 1996, 1997, 2002a). It has been demonstrated that electrical stimulation in the motor cortex induces an early, short-latency excitation in GPi neurons, followed by an inhibition and then a late, long-latency excitation (**Figure 1**; Nambu et al., 2000, 2002a; Tachibana et al., 2008). According to several pharmacophysiological data, the early excitation is most likely to be conveyed through the cortico-STN-GPi pathway (**Figure 1**; Nambu et al., 2000, 2002a; Tachibana et al., 2008). However, no direct evidence has as yet been available. By means of IT-mediated pathway-selective ablation, we have successfully proven the contribution of the hyperdirect pathway to the emergence of the early excitation. Here we introduce the detailed data on this issue.

## PREPARATION OF NeuRet VECTOR

**FIGURE 2:**
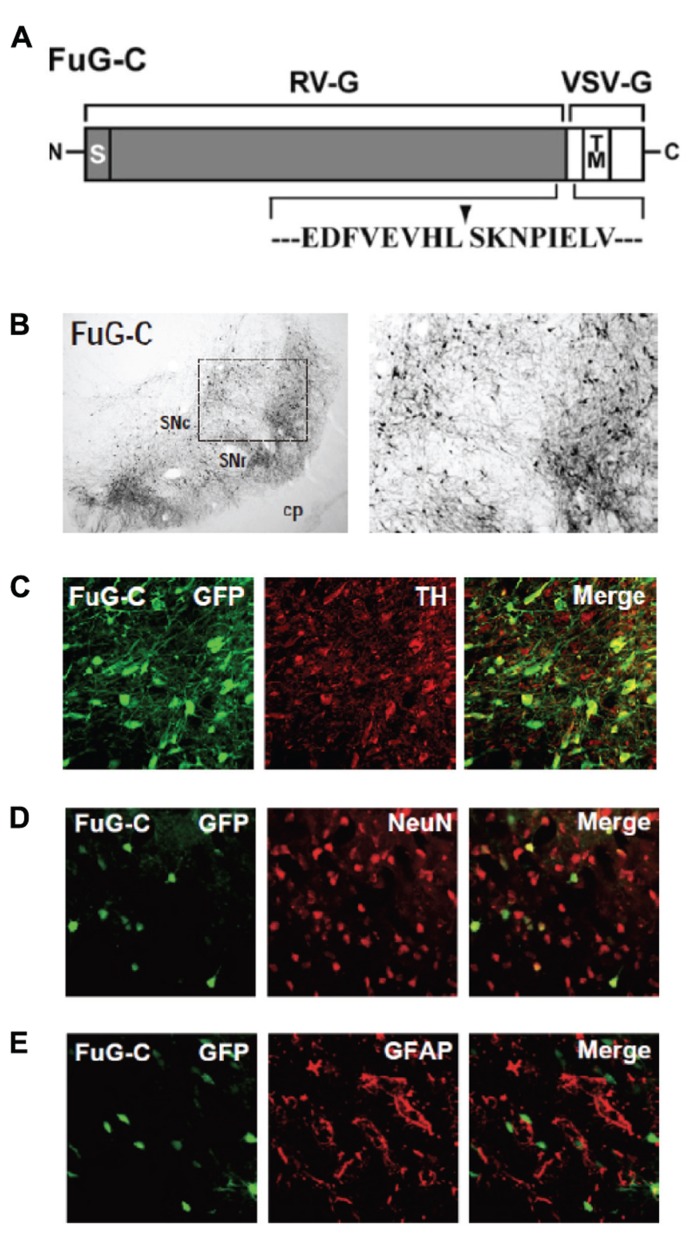
**(A)** Structure of fusion envelope glycoprotein of a neuron-specific retrograde gene-transfer vector (NeuRet vector). Fusion glycoprotein C type (FuG-C) is composed of the N-terminal segment of the extracellular domain of rabies virus glycoprotein (RV-G) and the C-terminal segment of the extracellular domain and the transmembrane (TM)/cytoplasmic domains of vesicular stomatitis virus glycoprotein (VSV-G). Amino acid sequences around the junction between the RV-G and VSV-G segments are shown. S, signal peptide. **(B)** Green fluorescent protein (GFP) immunostaining in the substantia nigra pars compacta (SNc). cp, cerebral peduncle; SNr, substantia nigra pars reticulata. **(C)** Double immunofluorescence staining for GFP and tyrosine hydroxylase (TH) in the SNc. **(D,E)** Double immunofluorescence staining for GFP/NeuN **(D)** or GFP/glial fibrillary acidic protein (GFAP; **E)** in the striatum.

We have developed a new vector system that permits NeuRet by pseudotyping the HIV-1-based lentiviral vector with fusion glycoprotein C type (FuG-C) consisting of a hybrid of rabies virus glycoprotein (RV-G) and vesicular stomatitis virus glycoprotein (VSV-G; Kato et al., 2011c). Interestingly, the NeuRet vector exhibits high efficiency of retrograde gene transfer into various populations of neurons, while it markedly reduces gene transduction into dividing cells, including glial and neural stem/progenitor cells, around the vector injection site. The NeuRet vector is composed of the N-terminal segment of the extracellular domain (439 amino acids) of RV-G and the C-terminal segment of the extracellular domain (16 amino acids) and transmembrane/cytoplasmic domains of VSV-G (**Figure 2A**). To verify the capability of the NeuRet vector for efficient retrograde gene transfer into the nigrostriatal pathway, we injected the vector encoding the green fluorescent protein (GFP) transgene into the striatum (caudate nucleus and putamen) of crab-eating monkeys. Intrastriatal injection of the NeuRet vector produced a large number of GFP-positive neurons in the nigra (**Figure 2B**). These neurons were immunostained for tyrosine hydroxylase, a key enzyme for dopamine biosynthesis (**Figure 2C**), indicating the transgene expression in the nigrostriatal dopamine neurons. Moreover, we assessed the extent of gene transfer with the NeuRet vector around the injection sites in the monkey striatum. The vector displayed a low level of gene transfer into neuronal cells, and the level of vector transfer into glial cells was also quite low in the striatum (**Figures 2D,E**). Therefore, the NeuRet vector mediates enhanced retrograde gene transfer into neuronal cells, whereas it reduces the efficiency of gene transfer into glial cells around the injection sites.

## SELECTIVE ABLATION OF HYPERDIRECT PATHWAY

The NeuRet vector expressing IL-2Rα was injected into electrophysiologically identified sites in the STN of Japanese monkeys. Following the vector injections into the STN, activity of GPi neurons was recorded in response to electrical stimulation in the supplementary motor area (SMA). In most of the GPi neurons in which certain responses were induced, we observed a triphasic response pattern consisting of an early excitation, a subsequent inhibition, and a late excitation, as previously reported in normal monkeys (see **Figure 1**; Nambu et al., 2000; Tachibana et al., 2008). This indicated that the vector injections into the STN did not affect cortically evoked responses of GPi neurons.

**FIGURE 3:**
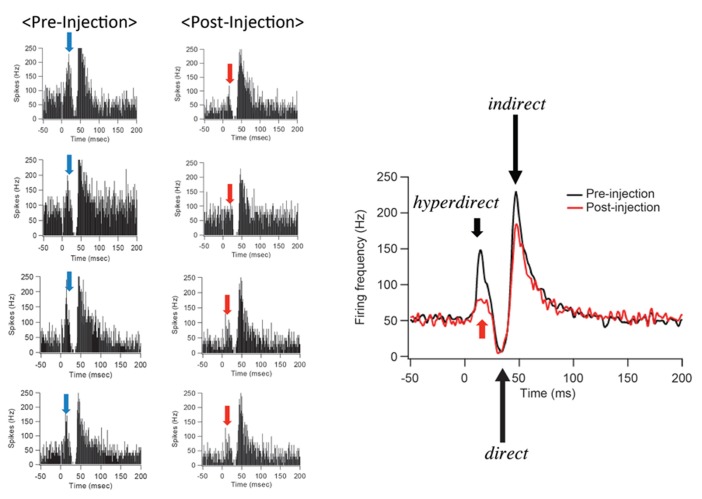
**Changes in GPi neuron responses after selective ablation of the hyperdirect pathway.** Examples of PSTHs (bin width of 1 ms, summed for 100 stimulus trials) before (Pre-Injection) and after (Post-Injection) immunotoxin (IT) injections into the supplementary motor area (SMA). Electrical stimulation in the SMA was given at time = 0. Note that the early excitation is diminished without either the inhibition or the late excitation affected.

**FIGURE 4:**
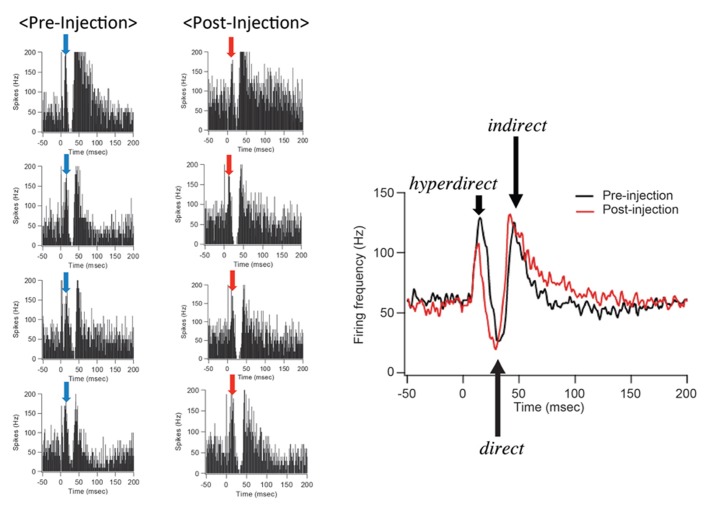
**GPi neuron responses to SMA stimulation in the control case in which no vector injections were made into the STN.** All conventions are as in **Figure 3**. Note that the response pattern of a GPi neuron is essentially the same before and after IT injections into the SMA.

After IT injections into the SMA, especially its arm region, neuronal activity in the GPi was recorded in response to the SMA stimulation. Many of the recorded GPi neurons exhibited a biphasic pattern, an inhibition followed by a late excitation without an early excitation (**Figure 3**). Compared with a control condition (before the IT injections), the amplitude of the early excitation was largely (by almost 90% of the control) reduced after the IT injections. On the other hand, the amplitude of the inhibition and the late excitation remained relatively unchanged, although the late excitation slightly decreased with no significant change (**Figure 3**). In addition, virtually no alterations were found in the latency of the inhibition or the late excitation, or the duration of the inhibition or the late excitation. In the control monkey in which no vector injections were made into the STN, the response pattern of GPi neurons on SMA stimulation was essentially the same before and after IT injections into the SMA (**Figure 4**). Thus, the IT injections into the SMA combined with the injections of the NeuRet vector expressing IL-2Rα into the STN abolished the cortically evoked early excitation in the GPi without affecting either the inhibition or the late excitation.

**FIGURE 5:**
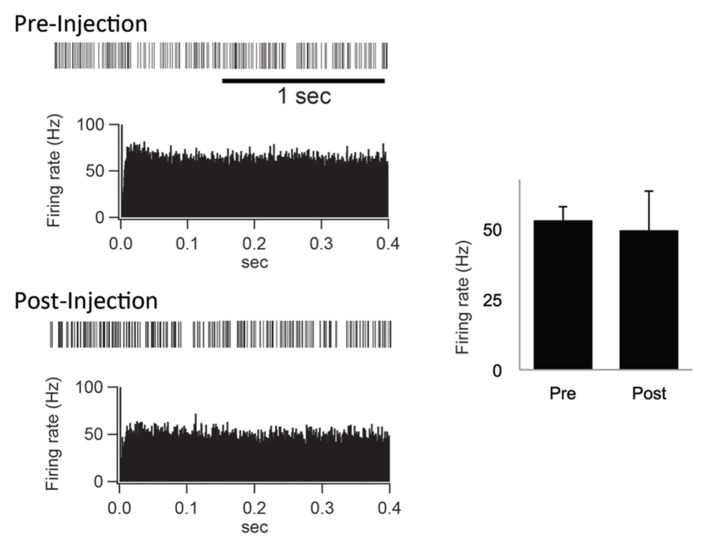
**(Left) Examples of spontaneous activities denoted by slow traces of digitized spikes and autocorrelograms (bin width of 0.5 ms) before (Pre-Injection) and after (Post-Injection) IT injections into the SMA.** (Right) Firing rates before (Pre) and after (Post) the IT injections.

Furthermore, the spontaneous firing rate and pattern were compared before and after the IT injections into the SMA. The spontaneous firing rate was left intact (**Figure 5**). Neurons in the GPi fired randomly at high frequency before the IT injections, and no apparent changes were observed after the IT injections (**Figure 5**). These results suggested that the firing rate and pattern of GPi neurons remained unchanged even after the removal of the cortico-STN projection.

**FIGURE 6:**
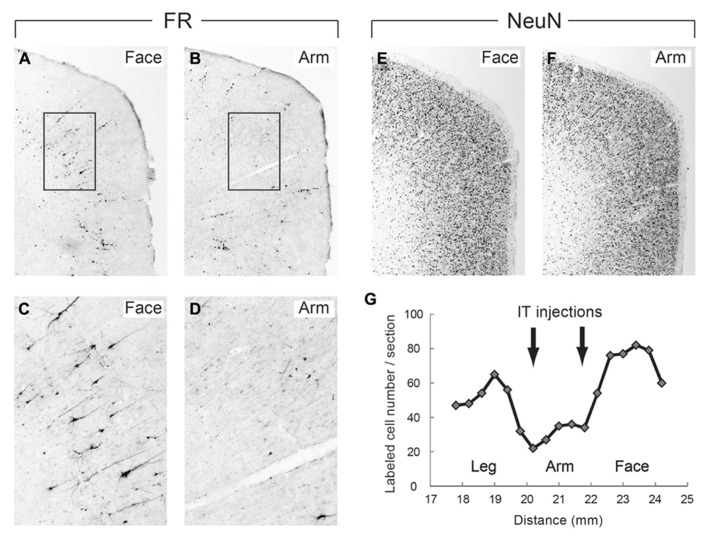
**(A,B)** Retrograde neuronal labeling in the face and arm regions of the SMA after Fluoro-ruby (FR) injection into the STN. **(C,D)** Higher-power magnifications of the rectangular areas in **(A,B)**. **(E,F)** NeuN immunostaining of the face and arm regions of the SMA. Note that the IT injections cause no marked tissue damage. **(G)** Distribution of FR-positive neurons in the face, arm, and leg regions of the SMA. Note that FR-positive neurons are so few in the arm region where IT injections were made (at two rostrocaudally distinct levels pointed to by arrows), as compared to those in the face and leg regions. Numerals on the abscissa represent the distance from rostrocaudal zero on the stereotaxic frame (equivalent to the interaural line).

In the monkeys subjected to the disappearance of the early excitation responding to the SMA stimulation, we performed retrograde and anterograde neuronal labeling by injecting Fluoro-ruby (FR) into the STN and biotinylated dextran amine (BDA) into the SMA. After the FR injection into the STN, retrogradely labeled neurons in the SMA were much fewer in the arm region where the IT injections were primarily aimed than in the face and leg regions (**Figure 6**). Moreover, immunostaining for NeuN revealed that the IT injections into the SMA caused no marked tissue damage (**Figure 6**). After the BDA injections into the SMA forelimb region, anterogradely labeled axon terminals were so largely decreased in the STN, as compared to the control case (data not shown). In remarkable contrast, dense terminal labeling from the SMA was seen in the striatum, especially the putamen, as in the control case (data not shown). These anatomical data clearly indicated that cortico-STN projection originating from the SMA arm region was selectively eliminated without affecting either the corticostriatal projection or the cortico-STN projections from SMA regions with other representations than the arm.

We did not closely examine behavioral changes to be produced after elimination of the hyperdirect pathway, because our study was primarily designed to develop a new methodological approach to selective removal of a given pathway. As far as our experimental conditions were concerned, no apparent motor abnormalities were observed.

## DISCUSSION

Taking advantage of the NeuRet vector that allows for highly efficient retrograde gene-transfer with improved neuron specificity (Kato et al., 2011c), we have established IT-mediated pathway-selective ablation in the primate brain. Here we have applied this technique to the hyperdirect pathway (Inoue et al., 2012). In conjunction with the direct and indirect pathways (for reviews, see Albin et al., 1989; Alexander and Crutcher, 1990), the hyperdirect pathway is known to be among the key pathways of the basal ganglia, being involved in motor information processing in the basal ganglia (Nambu et al., 1996, 2002a). This pathway connects the motor cortex to the GPi at short latency through the STN without relay at the striatum. When single neuron activity was recorded in the monkey GPi in response to electrical stimulation in the motor cortex, triphasic responses composed of an early (short-latency) excitation, an inhibition, and a late (long-latency) excitation were obtained. Based on the following pharmacophysiological data (see also Nambu et al., 2002a), it has been considered that the early excitation may be derived from the cortico-STN-GPi hyperdirect pathway: (1) Blockade of STN neuron activity by injection of the GABA_A_ receptor agonist, muscimol, there into abolished the early as well as the late excitation of GPi neurons (Nambu et al., 2000); (2) Blockade of glutamatergic input from the STN to the GPi by local injection of an ionotropic glutamatergic receptor antagonist diminished the early as well as the late excitation of GPi neurons (Tachibana et al., 2008).

For selective removal of the hyperdirect pathway, the NeuRet vector expressing IL-2Rα was injected into the STN, and, subsequently, IT was injected into the SMA in our experimental protocol. Our histological examination clearly indicated that cortical neurons in the arm region of the SMA projecting to the STN were selectively ablated. In such model monkeys, GPi neuron activity was recorded in response to electrical stimulation in the SMA. The SMA stimulation yields selective activation of SMA-recipient zones in the basal ganglia (Nambu et al., 2002b). It was found that out of the triphasic responses, only the early excitation was largely suppressed without either the inhibition or the late excitation affected. This indicates that IT-mediated tract targeting successfully eliminated the hyperdirect pathway selectively from basal ganglia circuitry. Our results define that the cortically driven early excitation of GPi neurons is derived from the cortico-STN projection. It has also been revealed that the firing rate and pattern of GPi neurons remain unchanged even after the removal of the cortico-STN projection. This implies that the cortico-STN projection conveys phasic activity changes from the SMA to the GPi, but does not contribute to maintenance of tonic activity of GPi neurons. In contrast to the early excitation, the inhibition in the GPi was not affected by the elimination of the cortico-STN projection, as it can be considered that the inhibition is mediated through the cortico-striato-GPi direct pathway (Tachibana et al., 2008). On the other hand, the late excitation in the GPi was slightly diminished though not significant. This late excitation is ascribable to the late excitation in the STN and is probably mediated by the cortico-striato-external pallidal segment (GPe)-STN-GPi indirect pathway. However, it has also been suggested that the late excitation in the STN is part of the prolonged excitation induced by the cortico-STN projection, which may explain a slight decrease in the late excitation in the GPi after the elimination of the cortico-STN projection (Tachibana et al., 2008). The SMA neurons giving rise to the cortico-STN projection are likely to issue axon collaterals to cortical and/or subcortical (other than the STN) regions. Thus, it cannot be ruled out that no such possible collateral projections may be affected by IT injected into the SMA.

According to the cortically driven triphasic response pattern elicited in GPi neurons, the hyperdirect pathway conveys excitatory signals from the motor cortex toward the GPi, bypassing the striatum, with shorter conduction time than signals via the striatum that arise from both the direct and the indirect pathways (see Nambu et al., 2000, 2002a). In favor of a dynamic “center-surround model” of basal ganglia function that was first proposed by Mink and Thach (1993), the functional role of the hyperdirect pathway has been implicated in the control of voluntary limb movements (see also Mink, 1996; Hikosaka et al., 2000; Nambu et al., 2002a). When a voluntary movement is about to be initiated by the motor cortical mechanism, a corollary signal conveyed through the cortico-STN-GPi hyperdirect pathway first inhibits large areas of the thalamic and cortical target structures that are related not only to a desired motor program, but also to other competing programs. Then, another corollary signal through the cortico-striato-GPi direct pathway disinhibits part of the thalamic and cortical target areas and releases the desired motor program alone. Finally, the third corollary signal conveyed by way of the cortico-striato-GPe-STN-GPi indirect pathway again inhibits the thalamic and cortical target areas extensively. By virtue of such sequential motor information processing, only the desired motor program is initiated, executed, and terminated at appropriate timings, whereas other competing programs are canceled. Thus, it is most likely that the hyperdirect pathway exerts a powerful excitatory effect on the GPi to suppress involuntary and unnecessary movements prior to the selected motor action. This notion is substantiated by the following issues: (1) Lesions or blockade of STN neuron activity induced involuntary movements, hemiballism (Carpenter et al., 1950; Hamada and DeLong, 1992; Nambu et al., 2000), suggesting that both the hyperdirect and indirect pathways might inhibit unnecessary movements; (2) According to functional magnetic resonance imaging studies using human subjects, the cortico-STN projection conveys stop signals to inhibit motor responses (Aron and Poldrack, 2006; Jahfari et al., 2011); (3) It is also suggested that the cortico-STN projection may inhibit automatic movements and switch to volitionally controlled movement (Isoda and Hikosaka, 2008).

Since no explicit motor abnormalities were seen in our experimental conditions, changes in the activity of GPi neurons in response to the cortical stimulation following a limited amount of elimination of the hyperdirect pathway may not be enough to elicit behavioral alterations. Accordingly, there is a need to determine the relationship between the extent of the elimination of the selected pathway and the expression of altered behaviors.

 The IT-mediated tract targeting achieves selective ablation of a given pathway in primates. This novel technique will provide a potent strategy to explore not only specific functional roles of individual pathways constituting a particular neural network, but also large-scale operative mechanisms underlying the entire network.

## Conflict of Interest Statement

The authors declare that the research was conducted in the absence of any commercial or financial relationships that could be construed as a potential conflict of interest.
